# Diagnostic Challenges of Demodicosis in a 65-Year-Old Man: A Case of Chronic Pruritus

**DOI:** 10.7759/cureus.86764

**Published:** 2025-06-25

**Authors:** Lucero de Maria Moran, Sarahi Carrillo, Aide Berenice Aguirre, Georgina Alejandra Martinez, Wendy Alejandra Castañeda

**Affiliations:** 1 Internal Medicine, Hospital Universitario de Torreón, Torreón, MEX; 2 General Surgery, Hospital Universitario de Torreón, Torreón, MEX

**Keywords:** chronic pruritus, demodex folliculorum, demodicosis, immunosuppression, ivermectin, metronidazole, nasal pruritus, topical corticosteroids

## Abstract

Demodicosis is a chronic skin disease characterized by pruritic erythematous macular lesions, primarily affecting the facial area, and caused by follicular mites of the genus *Demodex*. An imbalance in the immune system, such as immunosuppression or prolonged use of topical corticosteroids, may promote an increase in mite population, triggering an inflammatory and clinical skin response. Demodicosis is commonly associated with chronic pruritus, inflammatory lesions, and dermatological alterations such as irritant dermatitis and erythema in friction zones. Treatment remains challenging due to the lack of consensus on the most effective therapeutic approach. In this context, drugs such as metronidazole and ivermectin, both in topical and oral formulations, have shown efficacy in symptom control and in reducing *Demodex* mite infestation. This case report describes a 65-year-old male with a clinical history of generalized chronic pruritus and persistent cutaneous lesions, who demonstrated significant improvement following combination therapy with metronidazole and ivermectin, underscoring the importance of proper management in the treatment of demodicosis.

## Introduction

Demodicosis is an ectoparasitosis caused by mites of the genus *Demodex*, primarily the species *D*. *folliculorum *and* D. brevis* [1]. *Demodex* spp. belongs to the family Demodicidae, class Arachnida, order Acarina. These mites, with an average length of 0.3 mm, are acquired shortly after birth and are considered part of the normal flora of the human pilosebaceous unit, predominantly inhabiting the face, scalp, and upper trunk [2]. In healthy individuals, they are typically found in low densities and do not produce clinical symptoms. However, when the host-parasite balance is disrupted, such as in cases of immunosuppression or sebaceous gland dysfunction, excessive proliferation of these mites can lead to clinical manifestations. The prevalence of *Demodex* infestation increases with age, particularly in individuals over 50 years old, due to age-related changes in immune function and sebaceous activity, which favor mite overgrowth.

## Case presentation

**Figure 1:**
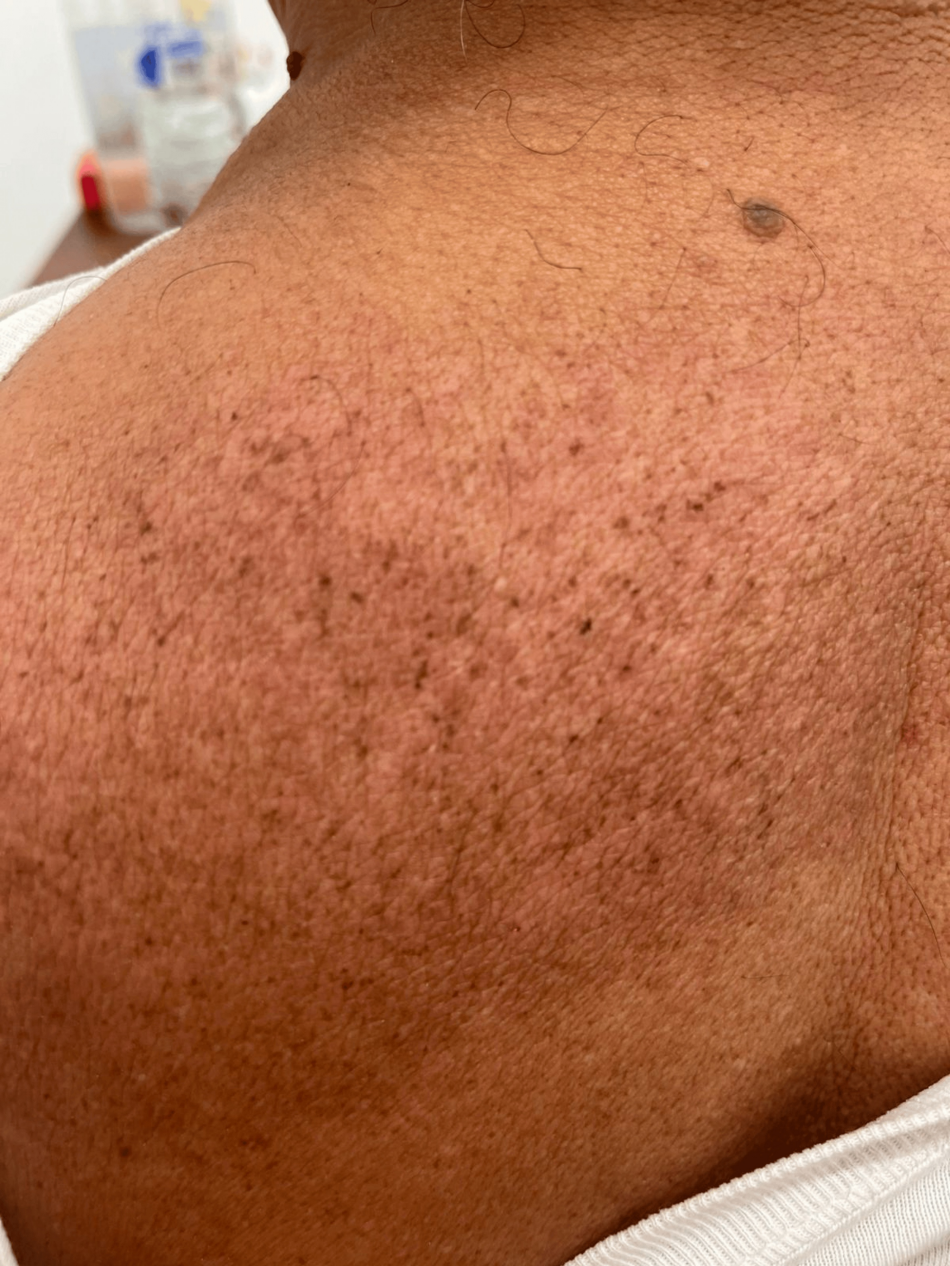
Skin lesion Initial dermatologic manifestations in the dorsal region of the patient.

We report the case of a 65-year-old male with no significant past medical history, who presented with chronic pruritus of 15 years’ duration, initially localized to the nasal region and progressively spreading to involve the trunk and intertriginous areas. The pruritus was described as persistent throughout the day, with nocturnal exacerbation and worsening under stress, leading to frequent sleep disturbances. The patient also reported sensations of formication, described as a crawling feeling beneath the skin, and chronic burning. Despite multiple treatments, including various oral antihistamines, topical corticosteroids, emollients, and alternative therapies, symptoms remained refractory. Dermatologic examination revealed several characteristic and secondary lesions. On the centrofacial region, especially the nasal dorsum and bilateral malar areas, there were ill-defined erythematous patches and thin plaques with fine scaling, associated with visible telangiectasias (Figure 1). Linear excoriations and scattered post-inflammatory hyperpigmented macules were noted on the nasal bridge, consistent with chronic scratching. There were no pustules, papules, nodules, or comedones in the facial area. The upper back and flanks showed multiple poorly demarcated erythematous macules and plaques with perifollicular scaling and greasy yellowish crusting, resembling seborrheic dermatitis. In these areas, signs of lichenification and excoriation were also present, along with numerous hyperpigmented macules, particularly over the parascapular regions.

**Figure 2:**
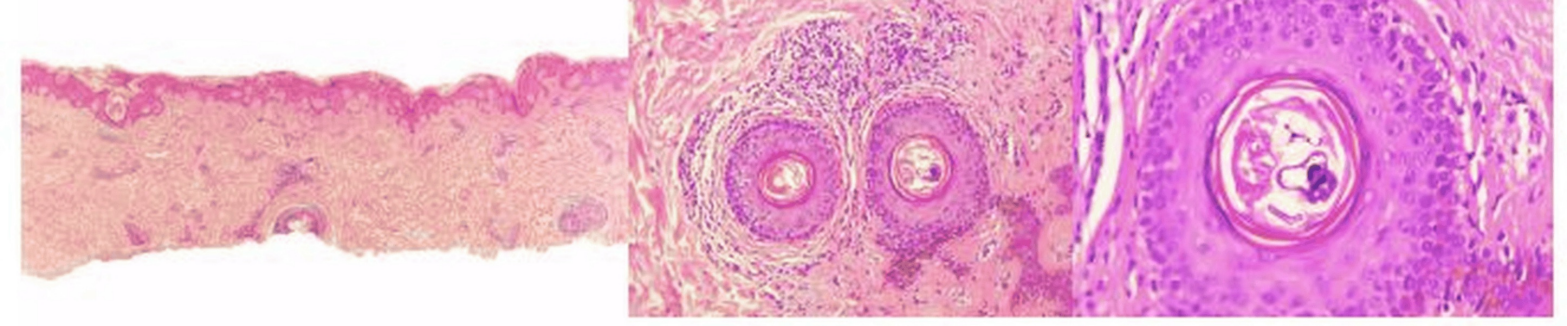
Dermatological histopathological report corresponding to a skin biopsy. The biopsy reveals a skin sample showing orthokeratotic epidermis with basket-weave stratum corneum and dermis containing superficial and perivascular lymphocytic infiltrates. There is perifollicular inflammation rich in lymphocytes without atypia, along with histiocytes. Within the follicular unit, structures consistent with Demodex mites are observed, identifiable by their characteristic elongated bodies and segmented exoskeletons. A special stain (PAS) was performed to rule out other microorganisms, yielding a negative result. The diagnosis is folliculitis due to *Demodex*.

Examination of the axillary, cervical, and lumbar folds revealed macerated erythematous plaques with epidermal thickening and superficial fissures, suggestive of chronic irritant contact dermatitis. No pustular lesions or signs of bacterial superinfection were found, and mucous membranes and nails were unaffected. Dermoscopy of the facial lesions, under polarized light, revealed perifollicular erythema, follicular plugs, and linear, translucent, white structures emerging from follicular openings, consistent with so-called “*Demodex* tails.” These features, while not specific, are considered indirect dermoscopic signs of *Demodex folliculorum* infestation. However, the dermoscopic images available were of low resolution and lacked standard annotation, limiting their interpretability and diagnostic value. Although the diagnosis of demodicosis is commonly established using non-invasive techniques such as standardized skin surface biopsy (SSSB) or superficial needle scraping (SNS) with quantification of mite density (>5 mites/cm² considered diagnostic), these methods were not available in our clinical setting. Given the chronicity, atypical dissemination, and refractoriness to standard therapies, a 4 mm punch biopsy was performed from a lesion on the upper back, where perifollicular inflammation and scaling were most prominent. Histopathological analysis revealed multiple *Demodex* mites within dilated hair follicles and sebaceous glands, associated with follicular hyperkeratosis, epidermal acanthosis, and a moderate perivascular lymphocytic infiltrate in the superficial dermis (Figure 2). While mite density was not quantified, the subjective burden was high. These findings, in conjunction with the clinical presentation and dermoscopic evidence, confirmed the diagnosis of disseminated demodicosis. The patient was treated with a combination of topical metronidazole 0.75% cream, applied twice daily, and two doses of oral ivermectin (200 μg/kg weekly). After three weeks, he experienced significant improvement in pruritus, erythema, and scaling, with partial resolution of lichenification and residual post-inflammatory pigmentation. He continues on regular dermatological follow-up with supportive barrier therapy and symptomatic control. 

## Discussion

Demodicosis is an ectoparasitic condition caused by mites of the genus *Demodex*, primarily *D. folliculorum *and* D. brevis.* These are microscopic mites, fusiform in shape with a transparent body, which inhabit human pilosebaceous follicles at low densities, forming part of the adult cutaneous microbiota [2]. They become pathogenic when their population increases abnormally, a condition more frequently observed in elderly individuals [3].

In this context, we describe an elderly patient with chronic generalized pruritus of insidious onset in the nasal region, progressively spreading. Initial management included various treatments, such as topical corticosteroids, with no clinical improvement. It is well documented that prolonged use of topical corticosteroids and other topical immunomodulators may induce local immunosuppression, facilitating *Demodex* proliferation on the facial skin [3].

The pathogenesis of demodicosis remains poorly understood and is still under investigation. Proposed mechanisms include mechanical follicular obstruction, follicular perforation with subsequent foreign body-type inflammatory response, cytokine and reactive oxygen species release that induces inflammation, and alterations in the cutaneous microbiome. Under physiological conditions, a dynamic balance exists between the host's immune system and mite populations, keeping them under control without clinical symptoms. However, local immunosuppression induced by external agents may disrupt this balance, leading to clinical manifestations [3]. 

This fragile host-parasite equilibrium can be disrupted by various predisposing factors such as systemic immunosuppression, diabetes mellitus, vasodilatory factors, and/or sebaceous gland hyperplasia [4]. *D. folliculorum *is preferentially located in facial follicles, including the cheeks, forehead, nose, temples, scalp, auricles, and eyelids [5]. As previously mentioned, prolonged topical corticosteroid use compromises cutaneous immunity, promoting ectoparasite overgrowth [3].

Histopathologically, demodicosis may present with follicular dilation due to infundibular infestation, follicular hyperkeratosis (which, together with mites, forms follicular spicules), presence of mites within follicles and/or sebaceous glands, and dense eosinophilic material surrounding them. The superficial dermis typically shows a peri-infundibular lymphocytic infiltrate, occasionally with histiocytes and multinucleated giant cells. In cases of follicular or glandular rupture, such as in granulomatous or pustular rosacea, an intense inflammatory reaction occurs, characterized by lymphohistiocytic infiltrate and multinucleated giant cells in a foreign-body pattern, surrounding the mites and forming suppurative granulomas [2].

The clinical and histological variability of demodicosis correlates with the degree of *Demodex* infestation [1]. Nevertheless, data from clinical trials remain limited, and there is no established consensus on a definitive treatment for inflammatory conditions associated with this parasitosis [6].

Systemic treatments include ivermectin and metronidazole. While clinical improvement in *Demodex*-associated lesions has been reported with these agents, consistent quantitative reductions in mite populations post-treatment have not been definitively demonstrated [4].

Regarding therapeutic approaches, a recent systematic review evaluated the efficacy and safety of various strategies for managing demodicosis, identifying metronidazole, both oral and topical, as the most commonly studied intervention in clinical trials. Notably, the combination of oral metronidazole with ivermectin demonstrated greater efficacy in normalizing demodectic density (Dd) than ivermectin monotherapy, suggesting a therapeutic synergy particularly beneficial in moderate-to-severe cases [6].

Pharmacologically, metronidazole is a synthetic nitroimidazole derivative with activity against anaerobic bacterial and parasitic infections. Its mechanism of action involves inhibition of nucleic acid synthesis via disruption of microbial DNA, explaining its efficacy in inflammatory cutaneous conditions associated with microbial or parasitic overgrowth [7]. Ivermectin, a macrocyclic lactone derived from *Streptomyces avermectinius*, is widely used for its potent antiparasitic properties and has also demonstrated efficacy in treating human demodicosis. Comparative studies have indicated that ivermectin has a faster onset of action and greater efficacy in reducing inflammatory skin lesions than metronidazole, supporting its use in initial management or cases with high parasite burden [7].

A key element in the pathophysiology of the exaggerated inflammatory response in demodicosis is the activation of Toll-like receptor 2 (TLR2) by *Demodex* antigens. This activation stimulates inflammatory cascades involving kallikrein-5 (KLK5) and the antimicrobial peptide cathelicidin LL-37. LL-37 promotes mast cell degranulation via the Mrgprx2 receptor, leading to neutrophil recruitment. These neutrophils, in turn, release additional LL-37, thus perpetuating the inflammatory cycle. Concurrently, fibroblasts secrete cytokines such as CCL19 and CXCL1, which attract CCR7+ T lymphocytes. Moreover, fibroblasts regulate prostaglandin D2 synthesis via prostaglandin D2 synthase (PTGDS), contributing to vasodilation and the erythema commonly observed in demodectic lesions [8].

Together, these mechanisms highlight the complex host-parasite interactions that drive the clinical manifestations of demodicosis and underscore the importance of timely recognition and appropriate management of this often underdiagnosed condition.

## Conclusions

Demodicosis is a cutaneous ectoparasitosis frequently observed in elderly patients, with a higher prevalence among those with immune dysfunction or a history of immunosuppressive treatments. This case illustrates an uncommon presentation of demodicosis with chronic, generalized distribution and atypical morphology. While *Demodex* mites are part of the normal skin microbiota, their uncontrolled proliferation can cause significant clinical symptoms, including pruritus, burning sensations, and inflammatory lesions. In this case, accurate diagnosis and appropriate treatment with ivermectin and metronidazole led to marked clinical improvement and enhanced quality of life, confirming the efficacy of this therapeutic combination in the management of demodicosis. However, due to the chronic nature of the disease and the potential for relapse, continuous monitoring of patients, particularly those with risk factors, is essential to ensure long-term management and control.

This case highlights the importance of considering demodicosis in the differential diagnosis of chronic, treatment-resistant pruritus, especially in elderly patients. The detailed description of the clinical presentation, diagnostic approach, and therapeutic response underscores key diagnostic clues, such as centrofacial erythema, follicular inflammation, and persistent paresthesias, that are often misattributed to allergic or inflammatory dermatoses. Histopathologic confirmation and a favorable response to combined ivermectin and metronidazole therapy emphasize the usefulness of antiparasitic treatment even in the absence of overt immunosuppression. Although histopathology is not the first-line diagnostic method for demodicosis, it serves as a valuable alternative in resource-limited settings or when non-invasive techniques are unavailable. This case also underscores the relevance of dermoscopy as a non-invasive diagnostic adjunct, provided high-quality, annotated images are used. Ultimately, an integrative clinical, dermoscopic, and histopathologic approach was essential for diagnosis and management in this challenging case. This report contributes valuable insight into an underrecognized condition at the intersection of dermatology and allergology, reinforcing the need for increased clinical awareness and a multidisciplinary management approach. 
